# Efficient butanol bioproduction from renewable lignocellulosic biomass by an integrated strategy of ternary deep eutectic solvent pretreatment and clostridial fermentation: toward complete utilization of reed straw

**DOI:** 10.3389/fmicb.2025.1670387

**Published:** 2025-09-10

**Authors:** Yu Shao, Cheng Zhang, Jiabin Wang, Fang Xie, Shijie Wang, Rongling Yang, Hongzhen Luo

**Affiliations:** School of Life Sciences and Food Engineering, Huaiyin Institute of Technology, Huai’an, Jiangsu, China

**Keywords:** green biomanufacturing, lignocellulose, enzymatic hydrolysis, butanol fermentation, *Clostridium acetobutylicum*, hydrochar

## Abstract

The main route for biobutanol production is anaerobic clostridial fermentation using corn and sugarcane as substrates. The high-price of raw materials in above processes largely limits its application as an advanced biofuel. The renewable lignocellulosic biomass is an ideal feedstock to produce butanol. However, the structure of lignocellulose is complicated which needs pretreatment to facilitate enzymatic efficiency and substrate fermentability. Herein, the green ternary deep eutectic solvent (DES) composed of choline chloride, succinic acid, and glycerol was synthesized. To improve DES pretreatment performance and reduce the viscosity of reaction system, water was added as co-solvent. Results indicate that xylan removal and glucan recovery were 61.7% and 93.6% when pretreating reed straw (RS) by DES/H_2_O (80:20, wt%) at 120 °C for 4 h. In this case, glucose yield reached 87.9% by cellulase after 72 h enzymolysis, which is 6.2-fold to untreated RS. Afterward, saccharification yield of treated reed was further elevated to 93.1% assisted by Tween 80 (100 mg/g-substrate). The reed hydrolysate was then applied as substrate for biobutanol production by *Clostridium acetobutylicum* ATCC 824, and approximately 6.5 g/L butanol and 8.8 g/L total solvents was obtained from the real hydrolysate. Finally, the enzymatic residues of reed were prepared to porous hydrochar materials via hydrothermal carbonization at 220 °C for 8 h. Adding 2.0 g/L hydrochar was verified for enhancing butanol titer to 11.5 g/L from synthetic medium containing typical pretreatment-derived inhibitors. In summary, this study provides an efficient butanol bioproduction route integrated with the complete valorization of renewable lignocellulosic biomass.

## 1 Introduction

The combustion of fossil fuels not only contributes to environmental pollution but also induces adverse effects such as global warming. Consequently, the identification of sustainable alternatives to fossil fuels has been considered as a critical research issue. Among biofuels, butanol has emerged as an efficient and environmentally sustainable substitute. Butanol exhibits advantageous properties including high energy density, low volatility, and low corrosiveness. Its compatibility with conventional hydrocarbon fuels positions it as a viable renewable substitute (Rafieyan et al., 2024). Biobutanol is mainly produced by clostridia via acetone-butanol-ethanol (ABE) fermentation, but several technical limitations have been encountered in this process.

In general, the main feedstock for biobutanol production is starch/sugar-rich substrates such as corn and sugarcane (Wang et al., 2023). The relatively high cost of raw materials is the critical challenge in industrial butanol production, necessitating the exploration of cost-effective and readily available alternatives. To this end, recent research has shifted to the use of lignocellulosic biomass as feedstock for clostridial fermentation (Luo et al., 2024). Zhang et al. (2023) explored the use of corn stover as a substrate and demonstrated its potential for efficient biobutanol synthesis. Similarly, the fermentation of sugarcane bagasse for biobutanol production using Clostridium beijerinckii has also been reported (Pratto et al., 2020). Common reed (Phragmites australis) is considered as a promising material for biorefinery due to its rapid growth and high biomass yield (Yang et al., 2022). It widespread distributes in China and about 7 × 10^7^ ton/year of reed straw are produced globally (Zhu et al., 2020). Additionally, the effectiveness of reed straw for butanol fermentation was investigated by organosolv pretreatment (Zhu et al., 2015). However, the complex structure of lignocellulosic biomass composed of cellulose, hemicellulose, and lignin, confers inherent recalcitrance to degradation. Consequently, efficient fractionation of lignocellulosic components constitutes a necessary step to biomass upgrading.

Deep eutectic solvent (DES) represent a new category of green solvent systems characterized by low vapor pressure, readily available raw materials, simple preparation, tunable properties, and exceptional recyclability (Tang W. et al., 2023). Compared with conventional methods, lignocellulose pretreatment via DES has been reported to generate less inhibitors, thereby enhancing enzymatic hydrolysis efficiency (Tang Z. et al., 2023). Chen et al. (2024) demonstrated that poplar pretreated with a ternary DES (benzyl triethyl ammonium chloride-ethylene glycol-FeCl_3_) at 130 °C for 4 h achieved enzymatic hydrolysis yield of 97.31%. Moreover, the incorporation of acidic solutions into DES has been reported to improve pretreatment efficacy by intensifying biomass delignification (Xie et al., 2024). It was reported that supplementing choline chloride-glycerol (ChCl-Gly) DES with H_2_SO_4_ during reed pretreatment effectively disrupt the recalcitrant cell wall, enhancing cellulase accessibility and fermentable sugar yields (Tang Z. et al., 2023). However, high viscosity of DES systems negatively affects mass transfer, posing limitations for industrial-scale applications (Zhang et al., 2024). Adding water can modulate the physicochemical properties of DES, reducing viscosity while maintaining pretreatment performance (Dai et al., 2015). Despite these advances, the synergistic effects of water and acid incorporation in ternary DES systems on biomass pretreatment and subsequent fermentation processes remain underexplored, necessitating further investigations.

Pretreatment of lignocellulosic biomass generates inhibitory compounds such as phenolics, which largely impede microbial growth and consequently reduce butanol yields (Luo et al., 2020). It has been suggested that biochar supplementation during fermentation enhanced microbial tolerance and improve bioalcohol production (Thunuguntla et al., 2024). Hydrothermal carbonization (HTC), recognized for its economically viable and environmentally benign applications, has garnered increasing attention as a viable strategy for effective biomass valorization (Ma et al., 2022). In HTC, biomass is utilized as feedstock and water serves as the liquid-phase reaction medium, converting raw materials into high-value-added products (primarily hydrochar) under controlled thermochemical conditions (150 °C–250 °C, 2–10 MPa) (Lv et al., 2023). As opposed to other thermochemical processes like pyrolysis (400 °C–600 °C), HTC has been demonstrated to consume less energy while maintaining operational efficiency (Zhao et al., 2022). The multifunctional applicability of hydrochar has been well-reviewed, including wastewater treatment and soil amendment (Oliveira et al., 2013). However, its role in modulating butanol fermentation remains underexplored, and its biochemical interactions with fermentative microorganisms remain to be investigated.

In this study, a ternary DES/water co-solvent system was established for the valorization of reed straw through pretreatment and enzymatic hydrolysis. The composition alterations in pretreated solid fractions and hydrolysis efficiency were evaluated. Pretreatment and hydrolysis conditions were carefully optimized. Comparative analysis of fermentable sugar yields under varying pretreatment conditions during high-solids enzymatic hydrolysis was performed under optimal conditions. The resultant reed hydrolysate was subsequently used as the substrate in butanol fermentation. Furthermore, hydrochar synthesized from enzymatic hydrolysis residue was evaluated for its potential to enhance butanol productivity, thereby achieving holistic valorization of lignocellulosic biomass.

## 2 Materials and methods

### 2.1 Materials and microorganisms

The reed straw was purchased from Suqian City, Jiangsu Province, China. It was crushed and filtered through a 40-mesh sieve (particle size approximately 0.4 mm). Cellulase (Cellic CTec3, filter paper activity of 100 FPU/mL) was purchased from Novozymes Biotechnology Co., Ltd. Choline chloride (98% purity) and other chemicals were purchased from Aladdin and Macklin Biochemical Technology Co., Ltd.

Butanol fermentation was performed using *Clostridium acetobutylicum* ATCC 824. Seed culture preparation involved inoculation in Clostridial growth medium (CGM) containing 30.0 g/L glucose (Luo et al., 2024), followed by incubation at 37 °C for 24 h in 100 mL anaerobic bottles. The chemical compositions of CGM contained 0.75 g/L KH_2_PO_4_, 0.75 g/L K_2_HPO_4_, 1.0 g/L NaCl, 0.017 g/L MnSO_4_⋅ 5H_2_O, 0.70 g/L MgSO_4_⋅ 7H_2_O, 0.01 g/L FeSO_4_⋅ 7H_2_O, 2.0 g/L (NH_4_)_2_SO_4_, 2.0 g/L L-asparagine, and 5.0 g/L yeast extract. When using CGM for butanol fermentation, the glucose concentration was adjusted according to the requirements.

### 2.2 Preparation of DES and hydrochar

The DES was produced by mixing choline chloride (ChCl), succinic acid (SA), and glycerol (Gly) at 90 °C under agitation for 2 h until a clear and uniform solution was formed. The molar ratio of ChCl-SA-Gly was maintained at 1:0.5:0.5. Hydrochar was conducted in a 250 mL vertical reactor (TGYF-B). A mixture containing 25.0 g reed enzymatic hydrolysis residue and 125.0 g deionized water was reacted at 220 °C for 8 h. After reaction, the reactor was cooled in a water bath until reaching ambient temperature. Subsequent solid and liquid phases were separated, followed by repeated rinsing of the solid fraction with deionized water until neutral pH (7.0) was attained. The solid was then dried in a oven at 55 °C for 24 h.

### 2.3 DES pretreatment and enzymatic hydrolysis of reed straw

Deep eutectic solvent pretreatment of reed straw was performed using a four-station parallel reactor (SLF-45-260) with a reaction volume of 45 mL. A solid-to-liquid ratio of 1:15–1:20 (w/w) was maintained in pretreatment system. An amount of acid (H_2_SO_4_/liquid of 1 wt%) were added in the reactor to compare pretreatment performance according to the requirements. Then, the reaction mixture was treated at 120 °C with 700 rpm for 3–4 h. Subsequently, the reaction mixture was transferred into a funnel for vacuum filtration. The solid fraction was repeatedly washed with deionized water until pH 7.0 was achieved, followed by drying at 85 °C for 10 h.

Enzymatic hydrolysis of the pretreated solids was conducted. A reaction system at 2 wt% solid loading, 0.5 g pretreated reed and 25 mL citrate-sodium buffer (50 mM, pH 4.8), was implemented in 100 mL flasks. Cellulase loading was maintained at 20 FPU/g substrate. In addition, the effect of Tween 80 addition (100 mg/g substrate) on enzymatic hydrolysis efficiency of DES-pretreated reed straw was investigated. For butanol production using pretreated reed biomass as substrates, the loading of solids was adjusted to 15 wt% while retaining the same enzyme dosage of 20 FPU/g substrate. Hydrolysis was carried out at 50 °C with 150 rpm for 72 h. Sampling intervals of 12–24 h were implemented, during which aliquots were centrifuged (10,000 rpm, 10 min) and the resultant supernatants were utilized for composition analysis.

### 2.4 Butanol fermentation

Butanol fermentation was conducted using the reed hydrolysate as substrate in 50 mL anaerobic bottles containing 20 mL fermentation broth inoculated with *C. acetobutylicum* ATCC 824. The enzymatic hydrolysate contained ∼85 g/L glucose, which was diluted to specified initial glucose concentrations (35–45 g/L). Nutrients were added and the initial pH was adjusted to 6.5 (Luo et al., 2024). Seed culture was centrifuged at 3,000 rpm for 3 min, and the cell inoculum was kept at 0.2 g/L. Static fermentation was carried out in a water bath at 37 °C for 72 h, with sampling intervals of 6–12 h. All collected samples were centrifuged (10,000 rpm, 10 min) prior to products analysis.

To evaluate the influence of hydrochar on butanol fermentation, a 5-L anaerobic bioreactor (BLBIO-5G) was used. In this case, four fermentations were conducted in CGM containing 60–75 g/L glucose with a working volume of 3.0 L, and the inoculum was 10% (v/v). The fermentation using CGM without hydrochar/inhibitors additions was used as the control group (batch #a). Some typical pretreatment-derived inhibitors including 0.2 g/L of vanillin and 0.2 g/L of 4-hydroxybenzaldehyde were added into broth at 0 h (batch #b). Meanwhile, 2.0 g/L of hydrochar was added into the broth at the beginning of fermentation to analyze its effects on butanol biosynthesis (batch #c). Finally, the pretreatment-derived inhibitors (i.e., 0.2 g/L of vanillin and 0.2 g/L of 4-hydroxybenzaldehyde) and 2.0 g/L of hydrochar were simultaneously added into the broth at 0 h (batch #d). The fermentations were conducted at 37 °C, 150 rpm for 48 h. During the cultivation, the pH was controlled at 5.0 by automatically feeding ammonia solution during acidogenesis. When butanol fermentation enters solventogenesis, the pH was not controlled (Luo et al., 2020). Collection of samples occurred at intervals of 6–12 h for the determination of products.

### 2.5 Structural characterization of reed straw

The microstructural characteristics of raw reed straw, enzymatic hydrolysis residue, and hydrochar were analyzed using a Gemini 300 scanning electron microscope (ZEISS, Germany). Fourier-transform infrared spectroscopy (FTIR, Thermo Nicolet iS20, United States) was employed to observe structural modifications with a range spanning of 4,000–400 cm^–1^ and a resolution of 4 cm^–1^. Thermogravimetric (TG) analysis of enzymatic hydrolysis residue and hydrochar was conducted via a thermal analyzer (Netzsch TG 209 F1 Libra, Germany) under high-purity nitrogen atmosphere (99.999%). Samples were heated from ambient temperature to 800 °C at a constant rate of 10 °C/min.

### 2.6 Analytical methods

Chemical composition analysis of DES-pretreated reed straw was performed following the NREL-TP-510-42618. The glucose concentration in liquid phase following enzymatic hydrolysis was quantified using an S-10 biosensing analyzer, and the concentration of xylose and total sugars was measured by HPLC system (LC-15C, Shimadzu, Japan). The yield of glucose, xylose, and total sugars was calculated by Equations 1–3.


(1) Glucose yield (%)=

$$\frac{\mathrm{Glucose}\,\mathrm{in}\,\mathrm{the}\,\mathrm{reed}\,\mathrm{hydrolysate}\,\left(g\right)\times 0.9}{\mathrm{Glucan}\,\mathrm{in}\,\mathrm{the}\,\mathrm{pretreated}\,\mathrm{reed}\,\left(g\right)}\times 100$$

(2) Xylose yield (%)=

$$\frac{\mathrm{Xylose}\,\mathrm{in}\,\mathrm{the}\,\mathrm{reed}\,\mathrm{hydrolysate}\,\left(g\right)\times 0.88}{\mathrm{Xylan}\,\mathrm{in}\,\mathrm{the}\,\mathrm{pretreated}\,\mathrm{reed}\,\left(g\right)}\times 100$$

(3) Total sugars yield (%)=

$$\frac{\mathrm{Total}\,\mathrm{sugars}\,\mathrm{in}\,\mathrm{the}\,\mathrm{reed}\,\mathrm{hydrolysate}\,\left(g\right)}{\left(\mathrm{Glucan}\,\mathrm{and}\,\mathrm{xylan}\right)\,\mathrm{in}\,\mathrm{pretreated}\,\mathrm{reed}\times 1.1\,\left(g\right)}\times 100$$

In ABE fermentation, the concentration of metabolites was analyzed using a GC1290 system equipped with an FFAP column (30 m × 0.32 mm × 0.5 μm) and flame ionization detector. Isobutanol was used as the internal standard for GC analysis. Statistical analysis was conducted using Microsoft Excel 2016, with significance threshold at *p* < 0.05.

## 3 Results and discussion

### 3.1 The composition changes of reed straw by DES/water co-solvent pretreatment

Pretreatment can effectively disrupt the compact lignocellulosic matrix, remove hemicellulose, and enhance cellulase accessibility. A ternary DES comprising choline chloride, succinic acid, and glycerol (molar ratio 1:0.5:0.5) was synthesized for the pretreatment of corn stover, achieving a glucan enzymatic hydrolysis yield of 94.7% (Luo et al., 2024). However, the high viscosity of DES system has constrained their broader adoption at the industrial scale. Previous studies have indicated that water incorporation into DES markedly decreases solvent density and viscosity (Zhang et al., 2024). To evaluate the efficacy of the ternary DES/water co-solvent in reed pretreatment, a comparative analysis of chemical composition of solid residues under varied pretreatment conditions was conducted. It has been reported that temperature escalation from 100 °C to 130 °C gradually increases xylan removal and delignification yield, while cellulose retention declines when temperatures exceed 120 °C (Xu et al., 2023a). In addition, 80 wt% aqueous DES solutions outperform pure DES in thermal stability, crystallinity modulation, delignification efficiency, and enzymatic digestibility (Chourasia et al., 2022). Therefore, the pretreatment temperature of 120 °C and co-solvent of DES/water (80:20, w:w) were selected (Table 1).

**TABLE 1:** Chemical composition analysis of reed samples after deep eutectic solvent (DES) pretreatment under different conditions.

Pretreatment	Pretreatment condition	S/L ratio (w/w)	Chemical composition (%)			Solid recovery (%)	Glucan recovery (%)	Removal (%)	
			Glucan	Xylan	Lignin			Xylan	Lignin
Entry 1	Raw reed straw	–	38.6 ± 1.9^f^	24.6 ± 1.9^a^	29.9 ± 0.5^b^	–	–	–	–
Entry 2	80 wt% DES, 3 h	1:15	49.1 ± 0.8^e^	15.6 ± 0.7^b^	26.8 ± 0.6^d^	66.5 ± 0.4^a^	84.5 ± 0.8^d^	57.7 ± 0.7^f^	40.4 ± 0.7^f^
Entry 3	80 wt% DES, 3 h	1:20	51.2 ± 0.9^d^	14.6 ± 1.1^b^	25.0 ± 0.7^e^	65.2 ± 0.6^b^	86.4 ± 0.6^c^	61.2 ± 0.9^d^	45.5 ± 0.9^d^
Entry 4	80 wt% DES, 4 h	1:15	55.3 ± 0.9^b^	15.2 ± 0.9^b^	23.0 ± 0.9^f^	65.3 ± 0.5^ab^	93.6 ± 0.6^a^	59.6 ± 0.8^e^	49.8 ± 0.7^a^
Entry 5	80 wt% DES, 4 h	1:20	53.2 ± 0.7^c^	14.7 ± 0.8^b^	24.8 ± 0.7^e^	64.3 ± 0.7^b^	88.6 ± 0.6^b^	61.7 ± 0.6^d^	46.7 ± 0.8^c^
Entry 6	80 wt% DES, 1 wt% H_2_SO_4_, 3 h	1:15	54.3 ± 0.5^bc^	10.8 ± 0.9^c^	28.1 ± 1.2^c^	60.1 ± 0.9^c^	84.5 ± 0.8^d^	73.5 ± 0.9^c^	43.6 ± 0.8^e^
Entry 7	80 wt% DES, 1 wt% H_2_SO_4_, 3 h	1:20	52.2 ± 1.0^d^	10.4 ± 0.5^cd^	30.8 ± 0.8^b^	59.9 ± 0.7^c^	80.9 ± 0.9^e^	74.6 ± 1.1^c^	38.4 ± 0.6^b^
Entry 8	80 wt% DES, 1 wt% H_2_SO_4_, 4 h	1:15	57.4 ± 0.8^a^	8.9 ± 0.8^de^	27.6 ± 0.6^cd^	57.6 ± 0.7^d^	85.7 ± 0.9^cd^	79.1 ± 1.0^b^	47.0 ± 0.7^c^
Entry 9	80 wt% DES, 1 wt% H_2_SO_4_, 4 h	1:20	54.3 ± 0.9^c^	8.1 ± 1.0^e^	34.5 ± 0.8^a^	60.2 ± 1.1^c^	84.6 ± 1.1^d^	80.2 ± 0.8^a^	30.7 ± 0.8^g^

The used DES was ternary ChCl-SA-Gly system at the molar ratio of 1:0.5:0.5. S/L ratio refers to solid/liquid ratio (w/w). The data are shown as mean ± SD and different superscript letters in the same column represent significant differences (*p* < 0.05).

The reed straw comprised 38.6% glucan, 24.6% xylan, and 29.9% lignin. Pretreatment with 80% DES for 3–4 h increased glucan content in the solid fraction from 38.6% to 49.1–55.3%, with glucan recovery rates of 84.5–93.6% (Entry 2–5). Meanwhile, xylan content decreased from 24.6% to 14.6–15.6% under these conditions. Extended pretreatment duration was shown to improve xylan removal efficiency from 57.7–61.2% to 59.6–61.7%. These results demonstrate that the efficacy of DES/water co-solvent system in disrupting the compact structure of lignocellulose and enriching glucan content in pretreated solids. Furthermore, pretreatment at a solid-to-liquid ratio of 1:20 outperformed that at 1:15 in the 80% DES system, with 4 h exhibiting superior performance to 3 h. This phenomenon is likely attributed to enhanced solvent penetration into cellulose fibers during extended durations, thereby promoting fiber swelling and structural loosening (Xu et al., 2020).

Chemically assisted pretreatment has been recognized as an effective strategy to enhance DES-based pretreatment efficiency (Tang Z. et al., 2023). Sulfuric acid, the most commonly used acid in lignocellulose pretreatment, is typically applied at concentrations of 1%–2% (w/w) (Ruan et al., 2024). In this study, 1% dosage (H_2_SO_4_/solvent of 1 wt%) was incorporated into the pretreatment system. The chemical compositions of pretreated reed are summarized in Table 1. The glucan content increased from 49.1–55.3% (Entry 2–5) to 52.2–57.4% (Entry 5–8) in the acid-assisted system, while xylan content decreased from 14.6–15.6% to 8.1–10.8%. Under these conditions, xylan removal efficiency was improved from 57.7–61.7% to 73.5–80.2%. It has been reported that supplementing choline chloride-glycerol DES with both water and sulfuric acid synergistically enhances pretreatment efficacy by intensifying hemicellulose hydrolysis and lignin solubilization (Chen et al., 2018). These findings confirm that H_2_SO_4_-assisted DES pretreatment constitutes an effective strategy for reed deconstruction.

### 3.2 Enzymatic hydrolysis efficiency of reed after DES/water co-solvent pretreatment

**FIGURE 1:**
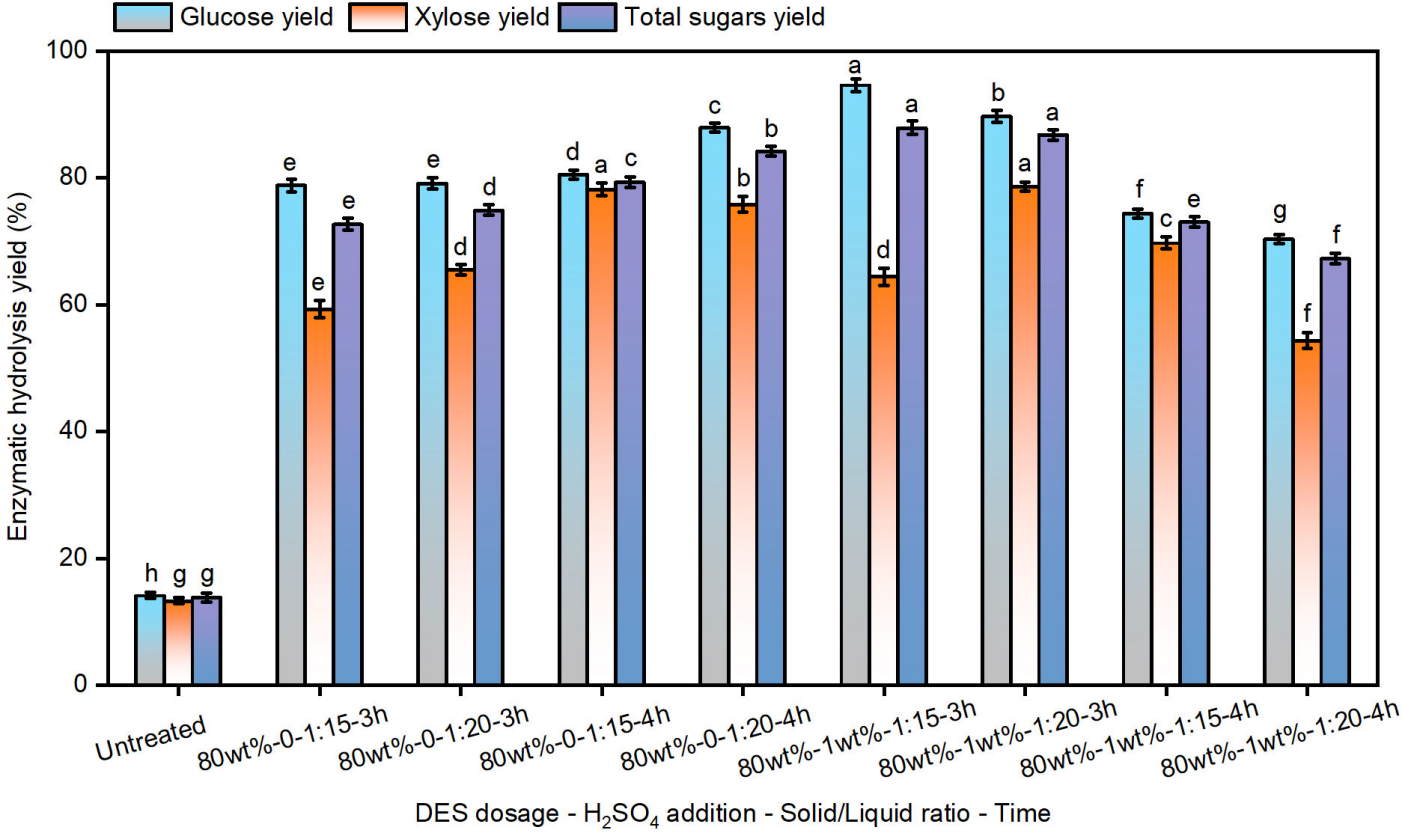
The enzymatic hydrolysis efficiency of reed straw after pretreatment using 80 wt% deep eutectic solvent (DES). The different superscript letters in the same column represent significant differences (*p* < 0.05).

Enzymatic hydrolysis efficiency serves as a critical metric for evaluating pretreatment efficacy and fermentable sugar production (He et al., 2023). Studies indicate that water addition to DES system facilitates carbohydrate conversion into monosaccharides (Xu et al., 2023b). The enzymatic hydrolysis of pretreated solids was conducted (Figure 1). When raw reed was used as the substrate, the yields of 14.1% glucose and 13.3% xylose were achieved with cellulase at 20 FPU/g substrate, demonstrating the inherent recalcitrance of untreated biomass (Luo et al., 2022). In contrast, glucose yields of 78.8%–87.9% were obtained from reed pretreated with 80% DES, with incremental improvements observed under prolonged pretreatment durations and higher solid loadings. These findings are consistent with prior reports indicating that DES pretreatment enhances cellulase adsorption and substrate accessibility (Zhou et al., 2024).

To further improve fermentable sugar yields, optimization of pretreatment conditions remains essential. Chen et al. (2018) achieved a glucose yield of approximately 89% using an acidified aqueous DES (ChCl-Gly) under mild conditions for switchgrass pretreatment. The enzymatic hydrolysis efficiency of solids pretreated with 1% H_2_SO_4_ assisted 80% DES was analyzed as well (Figure 1). At a pretreatment of 3 h, H_2_SO_4_-assisted pretreatment significantly increased glucose yields to 89.7%–94.6% compared to the non-acidified system. However, extending the pretreatment time to 4 h contributed to a reduction in glucose yields, in agreement with recent findings due to the strong severity under acidic condition (Tang Z. et al., 2023). Furthermore, enzymatic hydrolysis efficiency was found to decline with increasing solid-to-liquid ratios in H_2_SO_4_-assisted DES-pretreated solids (Entry 6–9). This reduction may be attributed to carbohydrate over-degradation under extended pretreatment and elevated solid concentrations. Although H_2_SO_4_-assisted DES pretreatment improves glucose release, it also lowers solid recovery rates, indicating the need for additional strategies to enhance hydrolysis efficiency (Xu et al., 2020).

**FIGURE 2:**
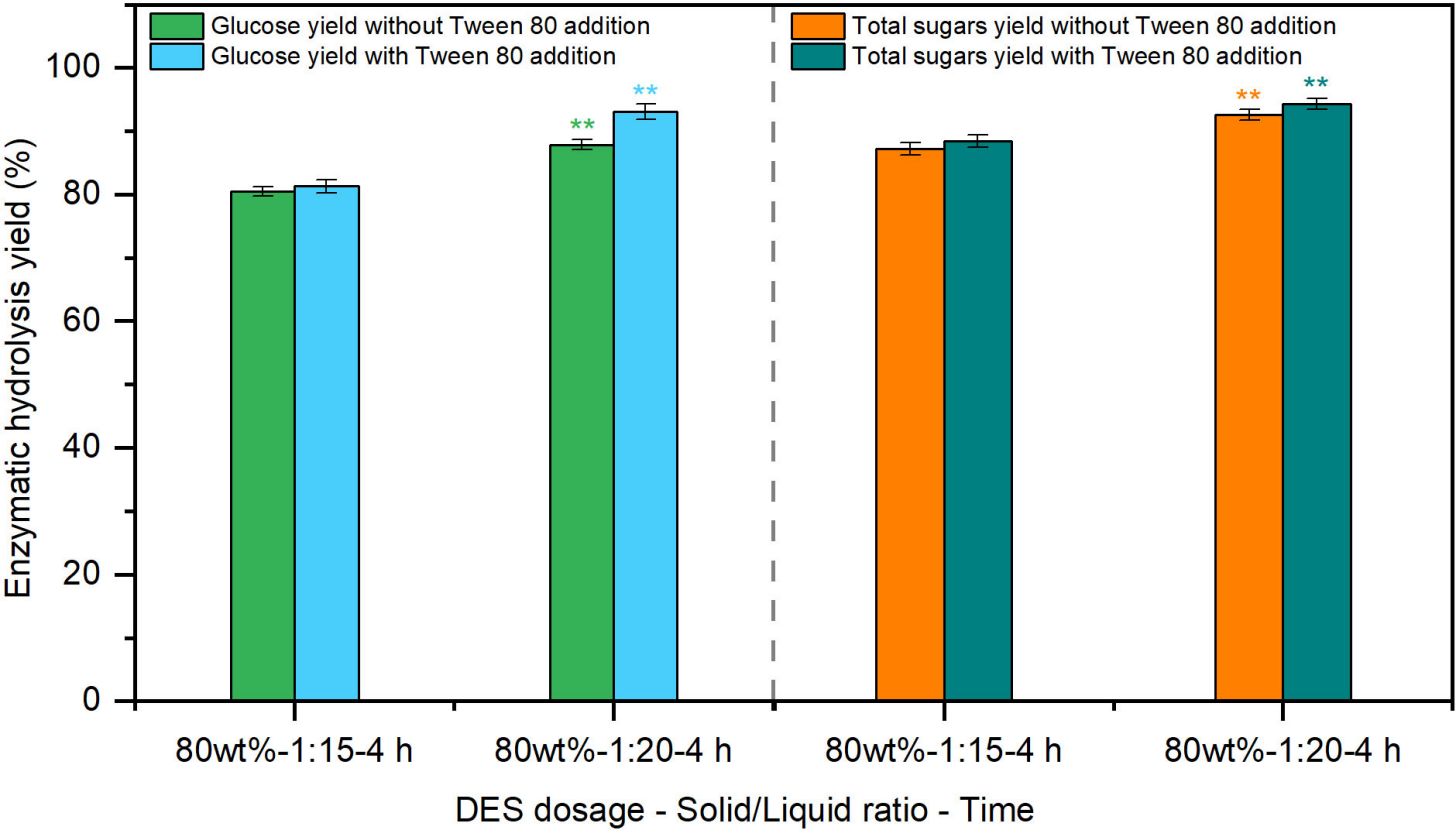
Effect of Tween 80 addition on hydrolysis yield of deep eutectic solvent (DES)-pretreated reed straw. The dosage of Tween 80 was 100 mg/g substrate. **p < 0.01.

Apart from pretreatment, the incorporation of additives during enzymolysis process is also considered an effective approach to maximize biorefinery efficiency (Sánchez-Muñoz et al., 2022). Among these, adding surfactants, such as Tween 80 and polyethylene glycol (PEG), were found to enhancing the enzymatic hydrolysis of lignocellulosic substrates (Li et al., 2012; Nogueira et al., 2022). The glucose yield from ChCl-formic acid DES-pretreated sugarcane bagasse reached 61%–82% by cellulase with the addition of Tween 80 at 100 mg/g substrate (Ling et al., 2021). Compared with other enzymatic auxiliaries, Tween 80 is a cost-effective additive, but its effect on enzymatic hydrolysis of ChCl-SA-Gly DES-pretreated reed has not been investigated. Therefore, the effect of Tween 80 at 100 mg/g substrate on the enzymatic hydrolysis of 80% DES-pretreated reed (Entry 5, Table 1) was assessed at a cellulase loading of 20 FPU/g substrate. According to Figure 2, glucose yields increased from 80.5–87.9% to 81.3–93.1% with Tween 80 addition, compared to surfactant-free controls. These findings suggest that Tween 80 supplementation serves as an effective process-intensification strategy for enhancing enzymatic hydrolysis yields in DES-pretreated reed samples. Therefore, the optimal conditions for producing fermentable sugars from reed using 80% DES pretreatment and enzymatic hydrolysis were determined as follows: pretreatment at 120 °C for 4 h with a solid-to-liquid weight ratio of 1:20 (Entry 5, Table 1), and enzymatic hydrolysis at 20 FPU/g substrate with Tween 80 addition of 100 mg/g substrate.

**FIGURE 3:**
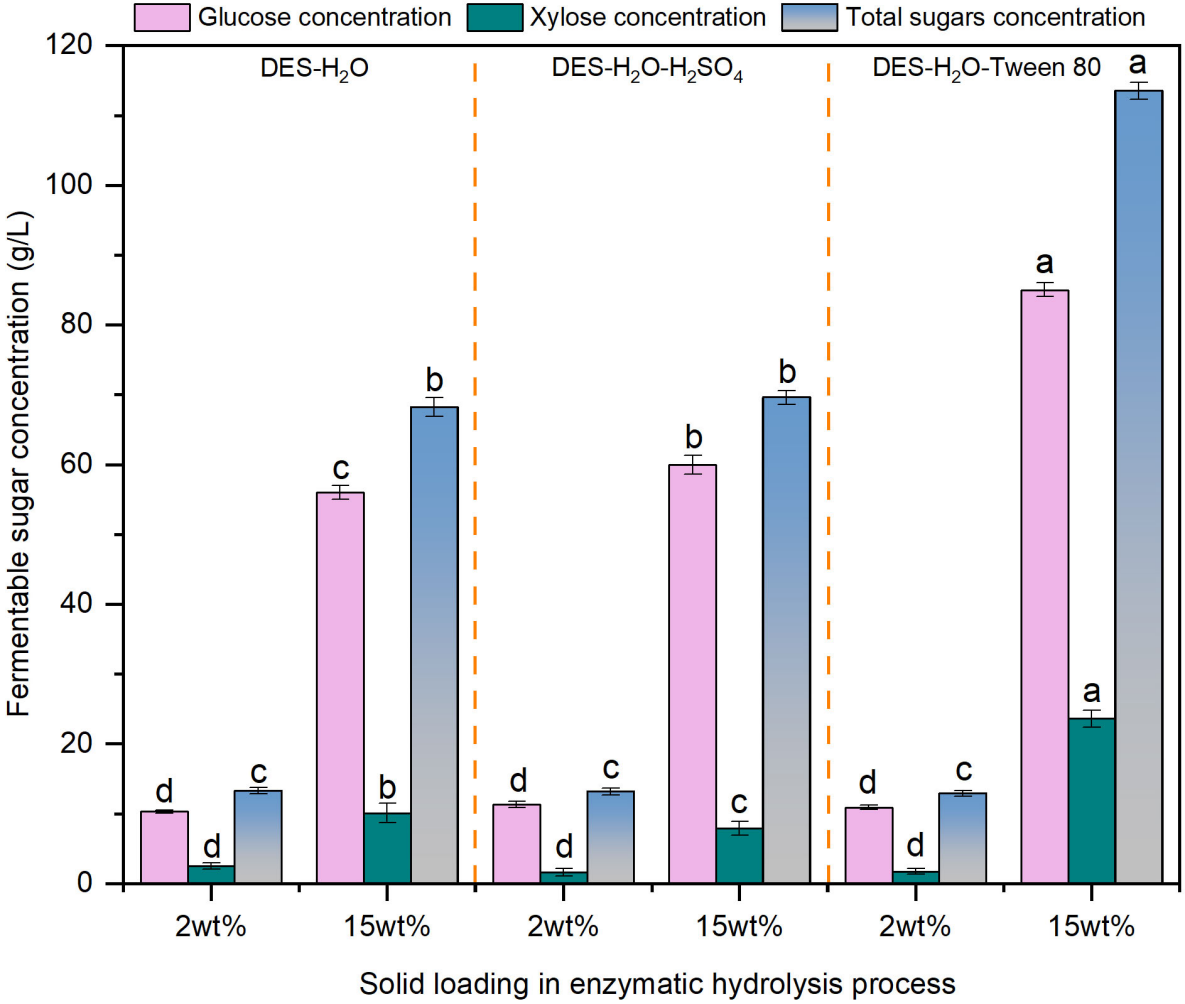
The fermentable sugars content in liquid fractions after 72 h enzymolysis using different solid loadings of deep eutectic solvent (DES)-pretreated reed straw. The different superscript letters in the same column represent significant differences (*p* < 0.05).

Enzymatic hydrolysis at high solids loading is essential for advancing economically feasible biorefinery technologies, and therefore, systematic evaluation of glucose concentration under elevated solids loadings is warranted (Sun et al., 2024). Based on compositional changes and hydrolysis efficiency, three optimal pretreatment conditions were selected for further evaluation. Comparative evaluation of glucose concentrations under high solids enzymatic hydrolysis (15 wt%) was conducted for the following groups: 80% DES pretreated (Entry 5), 1% H_2_SO_4_ assisted 80% DES pretreated (Entry 6), and Tween 80 supplemented enzymatic hydrolysis of 80% DES pretreated reed. As illustrated in Figure 3, the Tween 80 assisted system achieved the highest glucose concentration (85.0 g/L) from 80% DES pretreated reed (Entry 5), demonstrating the effectiveness of surfactant addition during high-solids hydrolysis. This result is similar to the findings which reported a glucose titer of 194.5 g/L produced from corn stover using a rhamnolipid surfactant (Qiao et al., 2024).

### 3.3 Butanol fermentation by *C. acetobutylicum* using reed hydrolysate as substrates

Butanol is extensively utilized as an industrial intermediate in chemical synthesis, plastics, and pharmaceuticals, while also recognized as an efficient liquid biofuel (Lin et al., 2023). Conventional acetone-butanol-ethanol (ABE) fermentation employs sugars or starch as substrates, yet the substantial substrate cost (accounting for ∼60% of total production expenses) significantly undermines its economic viability (Guo et al., 2022). Consequently, recent research has increasingly devoted to the utilization of lignocellulosic biomass as an alternative, renewable feedstock for butanol production. Xu et al. (2016) demonstrated the feasibility of this strategy, achieving a butanol concentration of 5.6 g/L using enzymatic hydrolysate derived from corn stover.

**FIGURE 4:**
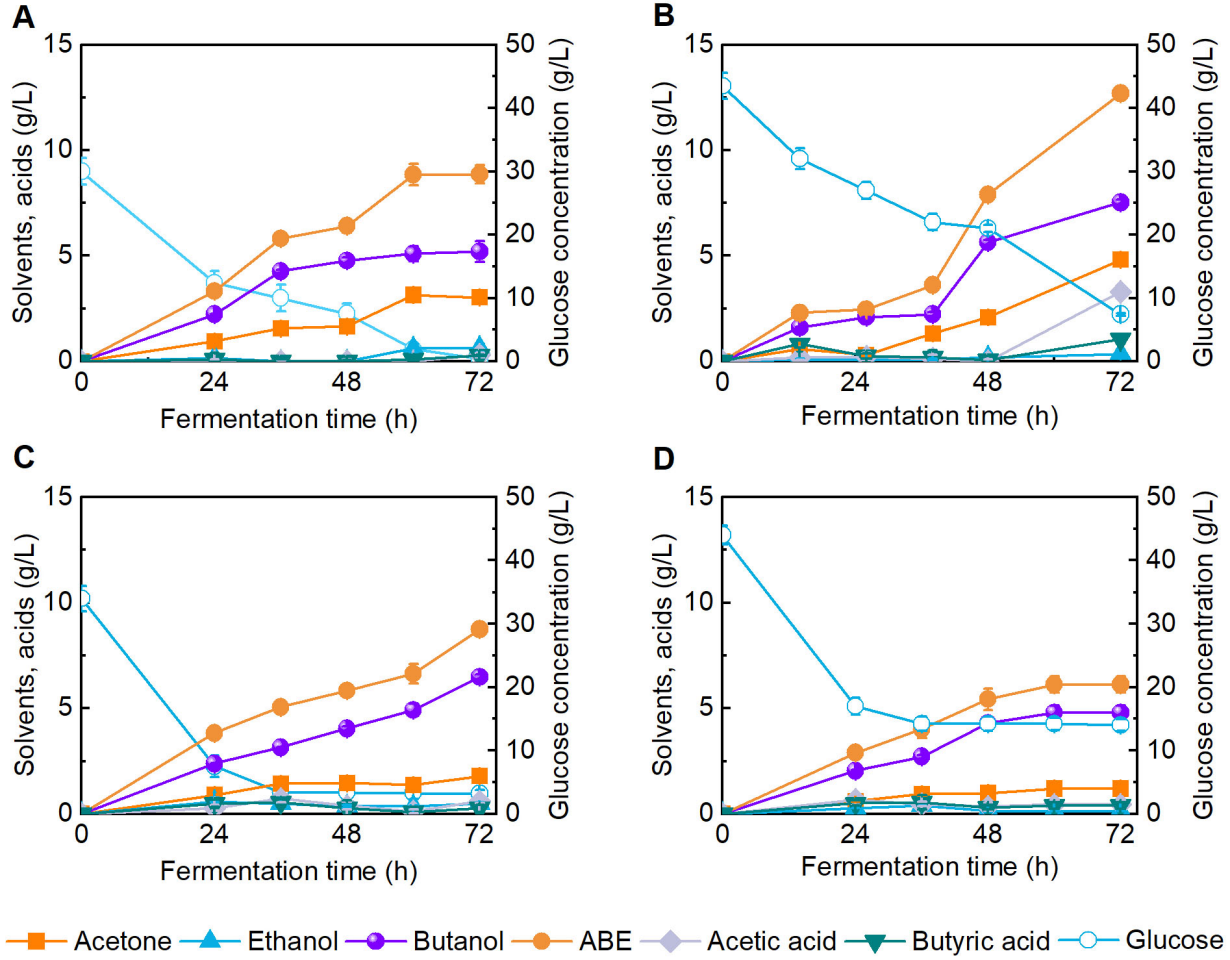
Butanol fermentation performance using Clostridial growth medium (CGM) and reed enzymatic hydrolysate as substrates. **(A)** CGM with 30 g/L of glucose; **(B)** CGM with 40 g/L of glucose; **(C)** Enzymatic hydrolysate with ∼30 g/L of glucose; **(D)** Enzymatic hydrolysate with ∼40 g/L of glucose.

To investigate the feasibility of using DES-pretreated reed as substrate for butanol production, we conducted butanol fermentation using enzymatic hydrolysates with varied sugar concentrations (Figure 4). It should be noted that, the treated reed straw was obtained from DES pretreatment (i.e., DES/H_2_O, 80:20) at 120 °C for 4 h (Entry 5, Table 1), and the solid loading for enzymatic hydrolysis was 15 wt% with cellulase of 20 FPU/g and Tween 80 addition of 100 mg/g substrate for 72 h. Figures 4A, B represent control butanol fermentations using CGM with 30 and 40 g/L glucose. The control group of 30 g/L glucose was basically exhausted after 72 h fermentation, and the final butanol titer reached 5.2 g/L. For the enzymatic hydrolysate containing 35.0 g/L glucose, near complete glucose depletion occurred after 72 h of fermentation, yielding a final butanol concentration of 6.5 g/L (Figure 4C). In contrast, the 45.0 g/L glucose hydrolysate exhibited incomplete glucose consumption and a lower butanol titer of 4.8 g/L after 72 h (Figure 4D). The above results indicate that the reed hydrolysate containing less than 45 g/L glucose was suitable for biobutanol production by clostridial fermentation. Similarly, an organosolv pretreatment using a mixture of ethanol and water has been developed to pretreat reed for biobutanol production (Zhu et al., 2015). As a result, 4.5 g biobutanol can be produced from 100 g of reed by *C. acetobutylicum* ATCC 824. Recently, the ternary DES was also used to pretreat corn stover to produce bioalcohol. Under mild conditions, 11.9 g/L of butanol was obtained via *C. acetobutylicum* fermentation (Luo et al., 2024). However, the DES system is inconvenient to operate for lignocellulose pretreatment due to its high viscosity. Therefore, compared with these studies, we added 20 wt% H_2_O to the ChCl-SA-Gly DES system to reduce its viscosity, making the operation more convenient.

### 3.4 Mass balance of butanol production based on DES/water co-solvent pretreatment

**FIGURE 5:**
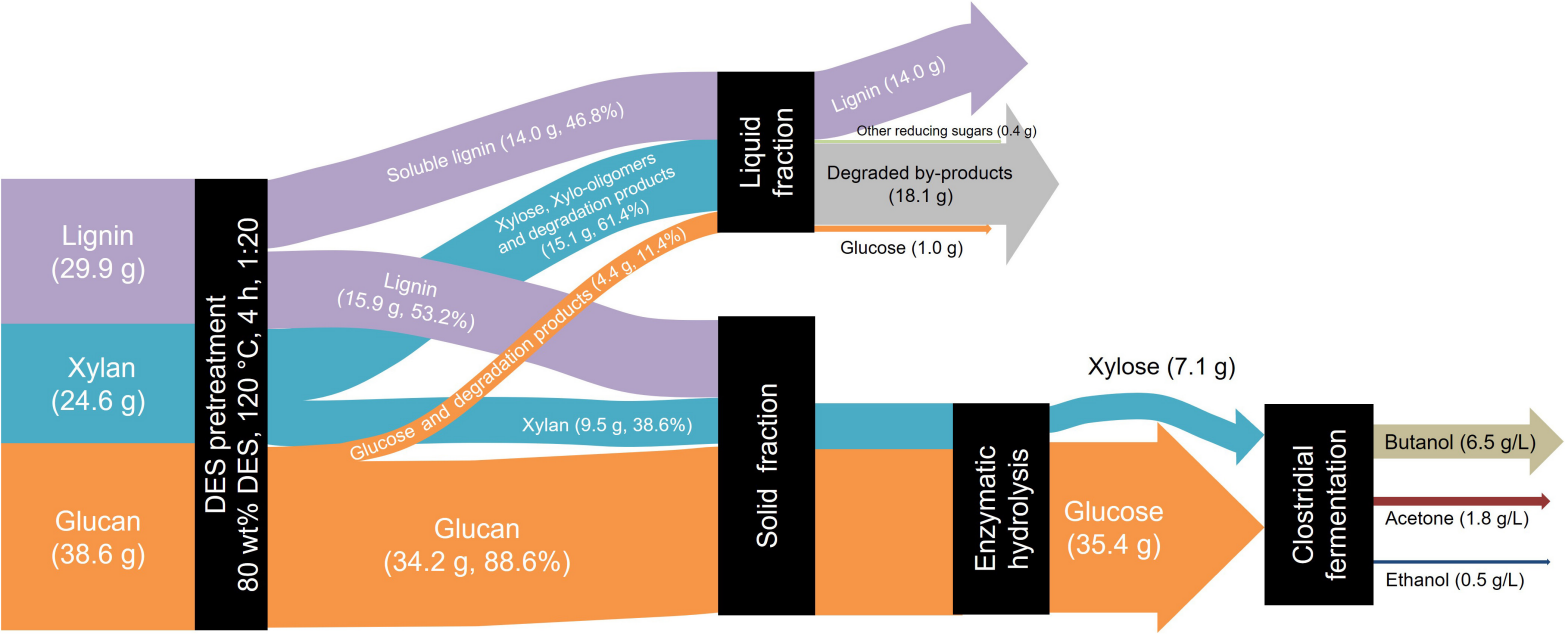
Mass balance of deep eutectic solvent (DES) pretreatment and enzymatic hydrolysis of reed straw for butanol fermentation.

The overall mass balance diagram under optimized pretreatment conditions is presented in Figure 5. A total of 100 g dry reed straw was pretreated with 80% DES at 120 °C for 4 h. Post pretreatment solid-liquid separation yielded a solid recovery rate of 64.3%, with the retained solids containing 34.2 g glucan, 9.5 g xylan, and 15.9 g lignin. Enzymatic hydrolysis was performed using cellulase (20 FPU/g substrate) supplemented with Tween 80 (100 mg/g substrate) under conditions of 50 °C and 72 h. The resultant hydrolysate was separated, diluted, and pH adjusted prior to fermentation at 37 °C for 72 h. The fermentation results reveal that the titer of butanol and total solvents (ABE) reached 6.5 and 8.8 g/L, respectively.

Previous studies have reported various strategies for butanol production using DES-pretreated biomass and corresponding enzymatic hydrolysates. These studies confirm that DES effectively deconstructs lignocellulose to enhance enzymatic hydrolysis, thereby providing sufficient fermentable sugars for butanol fermentation. Xu et al. (2016) employed a DES composed of choline chloride and formic acid to pretreat corn stover at 130 °C, achieving a butanol titer of 5.6 g/L from enzymatic hydrolysate (enzyme loading of 50 FPU/g). The total sugars production of 42.8 g/L and butanol titer of 9.5 g/L were obtained from rice straw by the combined DES (choline chloride, formic acid, and acetic acid) and Na_2_CO_3_ pretreatment (Xing et al., 2018). In contrast, about 0.5 g/L butanol was produced by *C. acetobutylicum* DSMZ 792 from ChCl-Gly DES-pretreated *Lactuca sativa* (Procentese et al., 2017). Compared to these methods, the 80% DES pretreatment strategy proposed in this study operates under milder conditions (120 °C) and requires a lower enzyme dosage of 20 FPU/g substrate for hydrolysis, demonstrating enhanced process sustainability and cost-effectiveness.

### 3.5 Characterization and analysis of hydrochar derived from enzymatic residues

Scanning electron microscope (SEM) was utilized to analyze microstructural features of raw reed, enzymatic residue using pretreated reed (Entry 5), and hydrochar. As shown in Supplementary Figure 1, raw reed samples exhibited an intact structure with a dense surface and limited porosity. Enzymatically hydrolyzed reed sample displayed structural disintegration characterized by increased surface wrinkling and fissure formation. Notably, hydrochar exhibited a highly porous morphology with distinct wrinkles, suggesting its potential to adsorb fermentation inhibitors and enhance microbial tolerance, thereby might facilitating *Clostridium* metabolic activity and improving butanol production efficiency.

**FIGURE 6:**
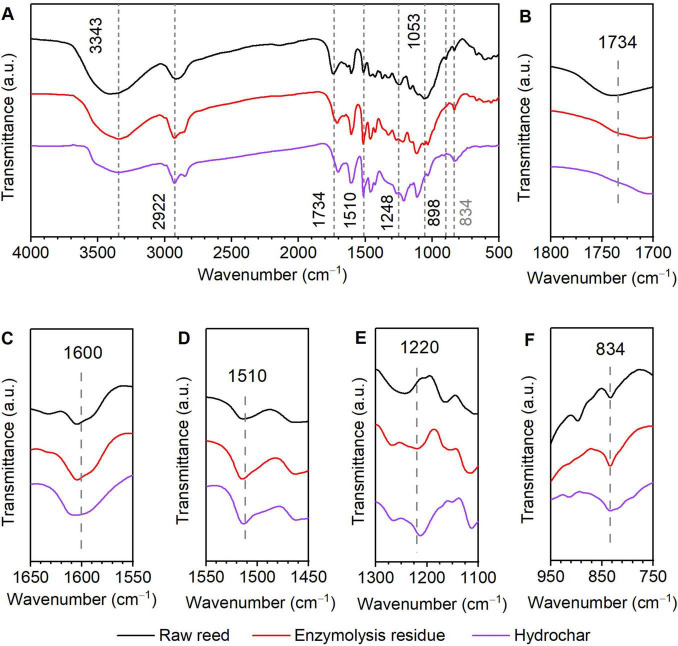
Fourier-transform infrared spectroscopy (FTIR) analysis of raw reed, enzymolysis residue, and hydrochar. **(A)** Wavenumber range of 4000–500 cm^−1^; **(B)** Wavenumber range of 1800–1700 cm^−1^; **(C)** Wavenumber range of 1650–1550 cm^−1^; **(D)** Wavenumber range of 1550–1450 cm^−1^; **(E)** Wavenumber range of 1300–1100 cm^−1^; **(F)** Wavenumber range of 950–750 cm^−1^.

Fourier-transform infrared spectroscopy was employed to investigate chemical structural alterations in raw reed, enzymatic hydrolysis residue, and hydrochar (Figure 6). The absorption band between 3,500 and 3,400 cm^–1^ is attributed to the skeletal vibrations of O–H stretching, while the absorption band between 2,930 and 2,900 cm^–1^ is due to the C–H stretching of methyl and methylene groups. The absorption band at 1,730 cm^–1^ corresponds to C = O stretching vibrations of acetyl groups in hemicellulose (Huang et al., 2022). As shown in Figure 6B, the disappearance of this band in enzymatic residue and hydrochar indicates substantial hemicellulose removal during enzymatic hydrolysis (Figure 1). The peak at 1,600 cm^–1^ (Figure 6C) is attributed to aromatic ring vibrations coupled with C = O stretching (aromatic skeletal vibrations) in lignin (Thoresen et al., 2021). The 1,510 cm^–1^ band (Figure 6D) represents skeletal vibrations of benzene rings in lignin molecules (Fan et al., 2023). A characteristic C–N stretching band at 1,220 cm^–1^ (Figure 6E), also associated with lignin, exhibited altered intensity in treated samples (Thoresen et al., 2021). Notably, the enhanced peak intensity at 834 cm^–1^ corresponds to deformation vibrations of C–H bonds in lignin aromatic rings (Figure 6F), suggesting structural modifications and lignin redistribution in enzymatic residue and hydrochar (Wang et al., 2024). Conversely, the disappearance of the 898 cm^–1^ band (Figure 6A), assigned to C–O–C stretching of β-glycosidic bonds in cellulose, confirms near-complete cellulose degradation after hydrolysis (Pancholi et al., 2023). In summary, the FTIR results show that there was good correlation between physiochemical analysis and experimental data.

**FIGURE 7:**
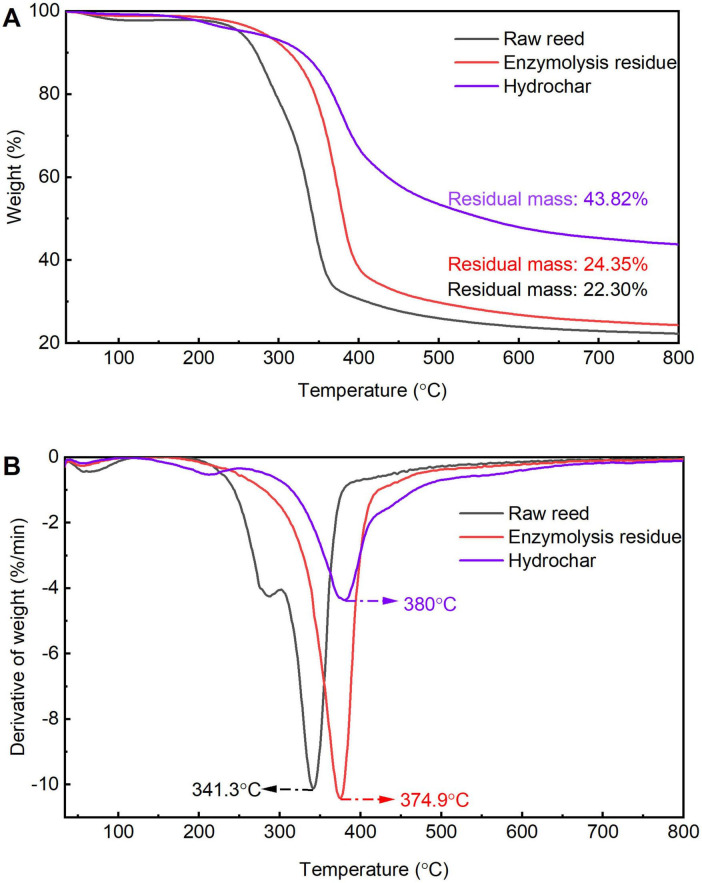
Thermogravimetric (TG) **(A)** and derivative thermogravimetric (DTG) **(B)** curves of raw reed, enzymolysis residue, and hydrochar.

Relevant studies have shown that biomass pyrolysis can be categorized into four distinct stages (Xiao et al., 2020). The first stage, termed the drying phase (< 100 °C), involves moisture evaporation, as evidenced by gradual mass loss on thermogravimetric (TG) and derivative thermogravimetric (DTG) curves. The second stage, the preheating phase (100 °C–*T*_0_, where T_0_ denotes the initial pyrolysis temperature), encompasses biomass depolymerization and glass transition. During the third stage (*T*_0_–*T*, with *T* representing the final pyrolysis temperature), volatile matter is released, the temperature range corresponds to the main stage of biomass pyrolysis, which is characterized by a significant change in the DTG curve. The final stage, the carbonization phase, involves slow decomposition of pyrolytic residues into char with minimal mass loss, reflected by a relatively flat DTG curve. In this study, thermogravimetric analysis (TGA) reveals distinct degradation profiles among raw reed straw, enzymatic hydrolysis residue, and hydrochar. As shown in Figure 7, the decomposition of raw reed was initiated at 253.8 °C, while enzymatic residue (334.9 °C) and hydrochar (340.2 °C) exhibited significantly higher onset temperatures. The first DTG peak of raw reed (250 °C–350 °C) corresponds to hemicellulose decomposition and decarboxylation of labile components (Wu et al., 2011). The absence of this peak in enzymatic residue and hydrochar suggests effective hemicellulose removal during pretreatment and carbonization (Figure 7). At elevated temperatures (350 °C–510 °C), degradation of thermally stable compounds, predominantly lignin, occurred (Soobhany et al., 2017). Enzymatic residue and hydrochar displayed peak maxima at higher temperatures compared to raw reed, indicative of enhanced thermal stability (Zhang et al., 2014). Notably, hydrochar exhibited substantially reduced peak intensity and slower mass loss rates relative to both raw reed and enzymatic residue. Residual mass results demonstrate that 22.3% char yield for raw reed versus 43.8% for hydrochar, further corroborating that hydrochar has superior thermal resistance.

### 3.6 Enhancing butanol fermentation performance with the addition of hydrochar derived from the enzymatic residues of reed straw

**FIGURE 8:**
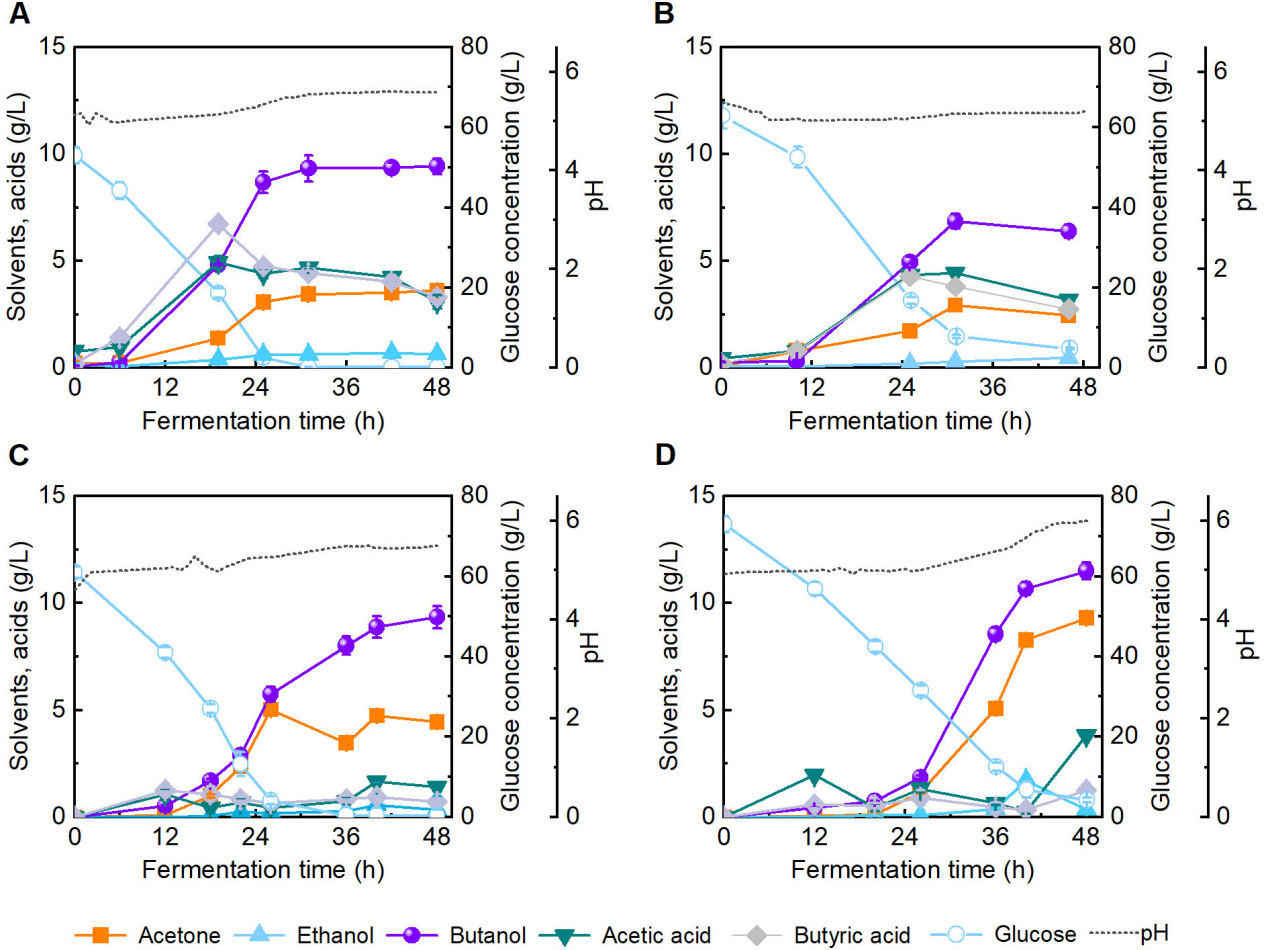
Fermentation performance of butanol in Clostridial growth medium (CGM) under different conditions. **(A)** Without addition of hydrochar and inhibitor; **(B)** Addition of 0.4 g/L pretreatment-derived inhibitors; **(C)** Addition of 2.0 g/L hydrochar; **(D)** Addition of 2.0 g/L hydrochar and 0.4 g/L pretreatment-derived inhibitors.

Carbonaceous materials have garnered growing interest owing to their notable porosity, thermal stability, and electrical conductivity (Khan et al., 2019). Biochar, a carbon-rich solid material, has been widely applied in microbial fermentation processes owing to its immobilization and adsorption capabilities (Zhang L. et al., 2023). Among various biochar synthesis techniques, HTC offers a low-temperature, low-pressure route for carbon material production, in contrast to high-temperature pyrolysis (400 °C–600 °C) (González-Arias et al., 2022). Although hydrochar has been applied in environmental remediation and agriculture (Oliveira et al., 2013), its impact on butanol fermentation remains underexplored. Concerns have been raised regarding the potential inhibitory effects of HTC-derived compounds on microbial viability, as well as residual pretreatment byproducts (Zhu et al., 2021). To investigate the biocompatibility of hydrochar in butanol fermentation, CGM was supplemented with 2.0 g/L hydrochar, and fermentation performance was evaluated (batch #c, Figure 8C). After 48 h of fermentation, butanol titer reached 9.3 g/L, showing no significant difference to the control group (batch #a, Figure 8A). The results demonstrate that hydrochar at the tested dosage exerts no observable inhibitory effect on butanol fermentation in CGM.

A series of compounds are generated during lignocellulose pretreatment, among which phenolic compounds exhibit the most pronounced inhibitory effect on butanol fermentation. In our previous studies (Luo et al., 2020, 2021), the effects of various concentrations of phenolics on butanol fermentation by *C. acetobutylicum* ATCC 824 were systematically analyzed, and butanol titer was not largely reduced when the vanillin concentration was below 0.2 g/L. Herein, to assess the inhibitory mitigation capacity of hydrochar under stress conditions, the addition of 0.4 g/L phenolic compounds (0.2 g/L of vanillin and 0.2 g/L of 4-hydroxybenzaldehyde) was added into broth. As illustrated in Figure 8B (batch #b), fermentation in inhibitor supplemented CGM without hydrochar yielded a final butanol concentration of 6.4 g/L. In contrast, supplementation with 2.0 g/L hydrochar under identical inhibitor conditions elevated the butanol titer to 11.5 g/L after 48 h (batch #d, Figure 8D), representing 1.7-fold enhancement relative to batch #b. These results indicate the ability of hydrochar to improve butanol fermentation performance in inhibitor challenged systems. The observed enhancement is likely attributable to the adsorption capacity of hydrochar, which attenuates the toxic effects of phenolic inhibitors on microbial activity (Supplementary Figure 2). This structure-function relationship was further supported by SEM analyses (Supplementary Figure 1). In summary, this study systematically examined the impact of various pretreatment strategies on the enzymatic hydrolysis efficiency and subsequent butanol fermentation performance of reed straw. Furthermore, hydrochar derived from enzymatic hydrolysis residue was employed to enhance butanol fermentation, thereby achieving the integrated valorization of lignocellulosic wastes. In the future, a comprehensive techno-economic assessment (TEA) of the overall process remains necessary to validate its industrial feasibility and sustainability.

## 4 Conclusion

The ternary ChCl-SA-Gly DES was applied to pretreat reed straw for enhancing enzymatic hydrolysis and substrate fermentability. Adding 20 wt% H_2_O to ChCl-SA-Gly system was found to improve glucose yield to 87.9% when treating reed at 120 °C for 4 h. In addition, Tween 80 dosage of 100 mg/g further elevated glucose yield to 93.1% after enzymatic hydrolysis. Afterward, the real reed hydrolysates were used for biobutanol fermentation by *C. acetobutylicum*, and ∼6.5 g/L butanol was obtained with a volumetric productivity of 0.09 g/L/h. The enzymatic residues of reed were treated by hydrothermal carbonization (220 °C for 8 h) to prepare hydrochar. The structure characteristics of hydrochar were analyzed by SEM, FTIR, and TG technologies. Finally, the effectiveness of hydrochar on enhancing butanol biosynthesis was successfully verified, and up to 11.5 g/L butanol was obtained from CGM contained 0.4 g/L pretreatment-derived inhibitors. The results would provide some guidance for green biomanufacturing of butanol from lignocellulosic biomass.

## Data Availability

The original contributions presented in this study are included in this article/Supplementary material, further inquiries can be directed to the corresponding author.
