# Supplementary material for: Efficient butanol bioproduction from renewable lignocellulosic biomass by an integrated strategy of ternary deep eutectic solvent pretreatment and clostridial fermentation: toward complete utilization of reed straw
Source: Front Microbiol. 2025 Sep 10;16:1670387. doi: 10.3389/fmicb.2025.1670387 (PMC12459719; doi:10.3389/fmicb.2025.1670387)
Supplement: Supplementary file 1 [file Supplementary_file_1.docx]

Supplementary Material

# Supplementary Figures


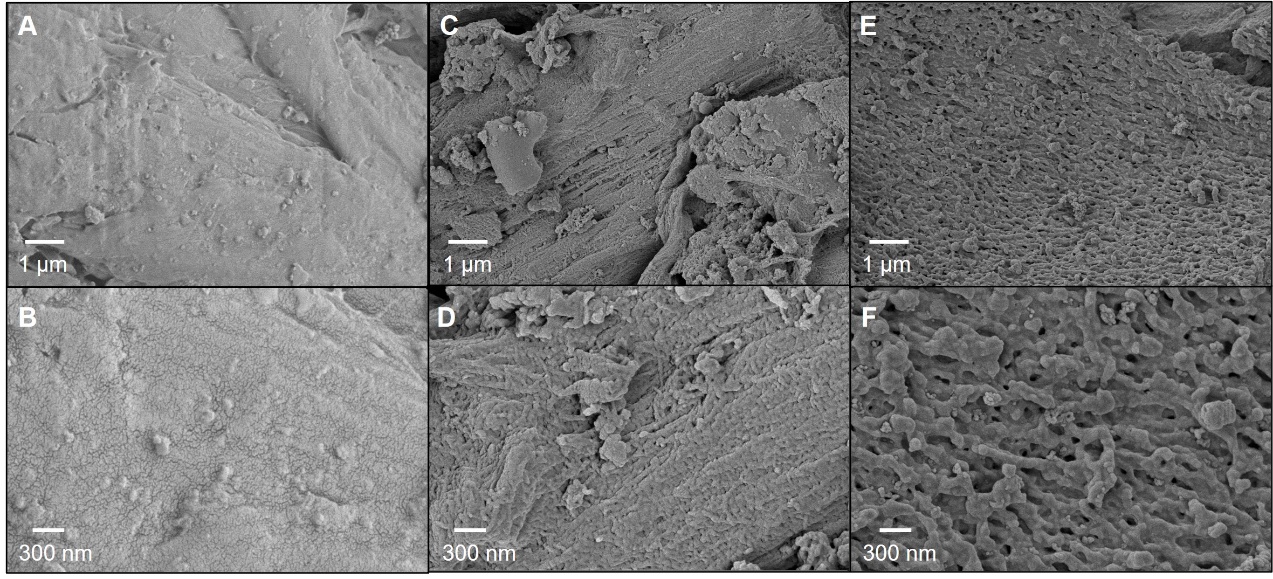


**Supplementary Figure S1.** SEM images of raw reed **(A-B)**, enzymolysis residue **(C-D)** and hydrochar **(E-F)**.


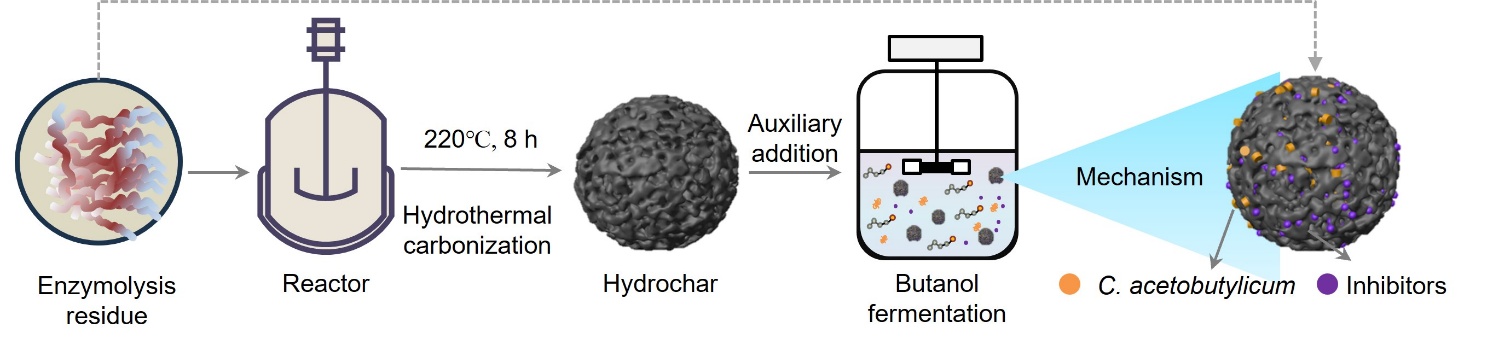


**Supplementary Figure S2.** Diagram of adding corn stover-derived hydrochar to improve butanol fermentation by *C. acetobutylicum*.
